# *Bacillus anthracis* Protective Antigen Shows High Specificity for a UV Induced Mouse Model of Cutaneous Squamous Cell Carcinoma

**DOI:** 10.3389/fmed.2019.00022

**Published:** 2019-02-12

**Authors:** Theo Crawford, Nicholas Fletcher, Margaret Veitch, Jazmina L. Gonzalez Cruz, Nicola Pett, Ian Brereton, James W. Wells, Mehdi Mobli, Yasvir Tesiram

**Affiliations:** ^1^Centre for Advanced Imaging (CAI), The University of Queensland, Brisbane, QLD, Australia; ^2^Australian Institute for Bioengineering and Nanotechnology (AIBN), The University of Queensland, Brisbane, QLD, Australia; ^3^Australian Research Council (ARC) Centre of Excellence in Convergent BioNano Science and Technology, Queensland Node, The University of Queensland, Brisbane, QLD, Australia; ^4^Faculty of Medicine, Translational Research Institute, University of Queensland Diamantina Institute, Brisbane, QLD, Australia

**Keywords:** skin cancer, Tumor Endothelial Marker 8, Protective Antigen, cutaneous squamous cell carcinoma, optical imaging, anthrax toxin receptors, Capillary Morphogenesis Gene 2

## Abstract

Squamous cell carcinoma (SCC) accounts for the majority of non-melanoma skin cancer related deaths, particularly in immunosuppressed persons. Identification of biomarkers that could be used to identify or treat SCC would be of significant benefit. The anthrax toxin receptors, Tumor Endothelial Marker 8 (TEM8) and Capillary Morphogenesis Gene 2 (CMG2), are endothelial receptors involved in extracellular matrix homeostasis and angiogenesis that are selectively upregulated on numerous tumors. One method of targeting these receptors is Protective Antigen (PA), a protein produced by *B. anthracis* that mediates binding and translocation of anthrax toxins into cells. PA targeted toxins have been demonstrated to selectively inhibit tumor growth and angiogenesis, but tumor selectivity of PA is currently unknown. In this work fluorescently labeled PA was shown to maintain receptor dependent binding and internalization *in vitro*. Utilizing a human papillomavirus transgenic mouse model that develops cutaneous SCC in response to ultraviolet irradiation we identified tumor uptake of PA i*n vivo*. The intravenously administered PA resulted in tumor specific localization, with exclusive tumor detection 24 h post injection. *Ex vivo* analysis identified significantly higher fluorescence in the tumor compared to adjacent healthy tissue and major clearance organs, demonstrating low non-specific uptake and rapid clearance. While both TEM8 and CMG2 were observed to be overexpressed in SCC tumor sections compared to control skin, the intravenously administered PA was primarily co-localized with TEM8. These results suggest that PA could be systemically administered for rapid identification of cutaneous SCC, with potential for further therapeutic development.

## Introduction

Non-melanoma skin cancer (NMSC) has the highest incidence rate of all cancers, higher than all other cancers combined (1). Although NMSCs are not generally associated with high mortality, due to their extremely high incidence they represent a significant financial burden (2). Squamous Cell Carcinoma (SCC) accounts for ~20 % of NMSCs but is responsible for the majority of NMSC related deaths due to potential metastatic spread if untreated (3), particularly in immunosuppressed persons (4, 5), where organ transplant recipients have a 65–100 fold increase in SCC incidence compared to the general population (6). Identification of potential biomarkers of SCC that can be used for targeted therapies and rapid non-invasive diagnosis is needed, particularly for advanced and metastatic cases where current treatments fail (4).

Protective Antigen (PA), a protein produced by *B. anthracis*, has been demonstrated to be a potential option for targeted delivery of drug molecules (7) or toxins (8) to tumors. PA is a component of the anthrax toxin facilitating the translocation of toxin enzymes into the cytosol through highly specific and selective interactions with two highly conserved anthrax toxin receptors known as Tumor Endothelial Marker 8 (TEM8) and Capillary Morphogenesis Gene 2 (CMG2) (9). TEM8 and CMG2 are endothelial cell surface receptors involved in extracellular matrix (ECM) homeostasis (10, 11) and angiogenesis (12, 13) that exhibit differential expression in healthy and tumor tissues. TEM8 directly interacts with the actin cytoskeleton (14) and components of the ECM, such as collagen I (15) and α3 VI (16). The expression of TEM8 does not affect cell proliferation or differentiation (17), nor is it necessary during normal adult angiogenic events (13, 18), but inhibition of TEM8 has been demonstrated to reduce tumor growth in mouse models (13). In patient derived samples of colorectal and gallbladder cancers TEM8 expression increases with cancer grade and clinical staging (19, 20), and has been associated with poor survival outcomes in breast cancer (21). CMG2 is involved in endothelial cell proliferation (12), adhesion (22), capillary formation *in vitro* (23), and collagen VI homeostasis *in vivo* (24). Expression of CMG2 has been inversely correlated to tumor progression in breast cancer (25) and soft tissue sarcoma (26). As TEM8 and CMG2 share ligands they are likely to have overlapping or complementary functions in response to changes in the ECM. Additionally, human and mouse anthrax toxin receptors are extremely conserved, an advantageous property for translational research of a targeting agent. The expression of the anthrax toxin receptors in tumors may inform on pathological changes to the ECM involved in angiogenesis, invasiveness, and metastatic potential.

However, despite PA targeted therapies showing promise in preclinical models (8, 27–32) there is currently no data that confirms tumor specific localization of PA *in vivo*. To determine whether PA can specifically target SCC tumors *in vivo* we recombinantly expressed PA and conjugated an amine reactive fluorescent dye for optical imaging of a HPV38E6E7-FVB transgenic mouse model. This model expresses oncogenes that interfere with cell survival pathways and promote cellular proliferation (33, 34) producing SCC in response to repeated UV irradiation, a mechanism that is highly relevant to human SCC development.

## Materials and Methods

### Recombinant Expression and Purification of *B. anthracis* Protective Antigen

BL21 *E. coli* was transformed with a pET-30b (+) plasmid containing a codon optimized Protective Antigen fusion protein with a C-terminal 12xHis tag preceded by a Tobacco Etch Virus cleavage sequence. The *E. coli* codon optimization, gene synthesis, molecular cloning, and sequence validation was performed by the University of Queensland Protein Expression Facility. Luria Broth media containing 50 μg/ml kanamycin was inoculated with the transformed *E. coli* and incubated at 37°C, with shaking (250 RPM). When the OD_600_ reached ~2–3, the temperature was reduced to 22°C. One milli meter isopropyl β-D-thiogalactopyranoside was added to the culture and incubated overnight with shaking. The cells were pelleted by centrifugation (10,000 × *g* for 15 min) and resuspended in Nickel Binding Buffer (50 mM sodium phosphate, 300 mM sodium chloride, pH 8) containing 200 μg/ml lysozyme, 1 mM dithiothreitol, 5 % (w/v) glycerol, and 0.1 % (v/v) Triton. The cells were sonicated on ice for 20 min (5 s on, 10 s off) and centrifuged (40,000 × *g* for 45 min). The supernatant was added to a pre-equilibrated 5 ml HisTrap FF column (GE life sciences). The column was washed with Nickel Binding Buffer containing 100 mM imidazole and eluted with Nickel Binding Buffer containing 250 mM imidazole. The imidazole was removed from the protein sample by buffer exchange using a 50 K molecular weight cut-off Amicon Ultra-15 Centrifugal Filter (Merck). one milli gram of Tobacco Etch Virus protease and 1 mM Dithiothreitol was added to the PA protein and incubated at 4 °C for 24–48 h. The cleaved protein was purified by reverse nickel affinity purification and the non-bound protein was buffer exchanged into 10 mM Tris, pH 8.0. The protein was added to a pre-equilibrated 5 ml DEAE anion exchange column (GE life sciences) and eluted with a 0–500 mM sodium chloride gradient over 200 ml. Fractions that contained the protein of interest were pooled and concentrated. Final purification of PA was performed by analytical size exclusion chromatography using a Superdex 200 Increase 10/300 GL column (GE life sciences).

### Fluorescent Dye Conjugation

Ten molar excess of Sulfo-Cyanine5 (Cy5) NHS ester (Lumiprobe) was dissolved in 25 μL of dimethyl sulfoxide solution, and diluted to 100 μL with water. Nine Hundred micro liter of recombinant PA protein (1–2 mg/ml) in 50 mM sodium phosphate buffer pH 8 was added to the dye solution, vortexed, and stored at 4°C overnight. The unreacted dye was removed using a 10K MWCO Amicon Ultra-4 Centrifugal Filter (Merck).

### Cell Line

The HPV38E6E7 SCC cell line, originating from tumors of UV-irradiated HPV38E6E7-FVB mice, was generated in-house (35) and grown in a modified F12 medium supplemented with Dulbecco's Modified Eagle Medium and Fetal Bovine Serum (FBS) in a 37°C incubator containing 5 % CO_2_.

### *In vitro* Binding Assay

HPV38E6E7 SCC cells were plated onto poly-L-lysine coated coverslips and blocked using Roswell Park Memorial Institute medium containing 20 % FBS. Cells were incubated with PA-Cy5 (15 nM) in media at 37°C for 30 min or at 4°C for 30 min, washed with phosphate buffered saline (PBS) and incubated in fresh media at 37°C for 30 min. The blocking experiment was performed by incubating the cells with unlabeled PA (30 nM) in media on ice for 30 min, washed with PBS and incubated with PA-Cy5 described as above. As a control, cells were incubated with free Cy5 dye (1 μM) in media on ice for 30 min, washed with PBS, and incubated in fresh media at 37°C for 30 min. After the final incubation, cells were washed with PBS, fixed in 4 % paraformaldehyde, and stained with 4′,6-diamidino-2-phenylindole (DAPI). Images were taken with a Nikon Ti/Spectral spinning disc confocal microscope, Plan Apo TIRF 60X Oil DIC H N2 objective, Andor Clara Dr-3222 camera, and NIS elements software. Integrated density of Cy5 fluorescence per cell was determined using maximum intensity projections of z-stacks using Image J FIJI software, cell boundaries were determined using cell autofluorescence.

### UV Irradiation of Mice

This study was carried out in accordance with the principles of the Basel Declaration and recommendations of Animal Care and Protection Act (2001) and the Australian code for the care and use of animals for scientific purposes (8th Edition). The protocol was approved by the University of Queensland Molecular Bioscience Animal Ethics Committee. An area of ~ 4 × 4 cm on the dorsal region of female HPV38E6E7-FVB mice was shaved periodically to remove fur. Sunblock was placed on the ears prior to irradiation to limit damage to ear skin. The awake animals were placed in a cage under a UV lamp and received a single exposure of UVB radiation three times a week starting at 120 mJ/cm^2^, gradually increased to 450 mJ/cm^2^ by day 140. UV dosing remained at 450 mJ/cm^2^ until characteristic SCC was observed (33). Visual characteristics include but are not limited to the formation of wart like growths, elevated growth with a central depression, or open sores. Female HPV38E6E7-FVB mice of similar age without UV irradiation were used as controls.

### Optical Imaging

Three weeks after the final UV treatment 75 μg of PA-Cy5 in PBS was administered intravenously via the tail vein and imaged using a Bruker Carestream *in vivo* MS FX Pro. *In vivo* images were taken prior to injection, 8, 24, and 48 h post injection. Animals were anesthetized with 3–5% isoflurane and allowed to recover at each imaging time point. The mice were sacrificed 48 h post injection, and the major organs were removed for *ex vivo* imaging. Fluorescence images were obtained using 7 excitation wavelengths (550, 570, 590, 600, 610, 620, and 630 nm), 10 s exposure, aperture (f stop) 2.8, field of view 190 mm, 2 × 2 binning, 700 nm emission filter. Reflectance (white light) images were obtained using 450 nm excitation wavelength, 0.5 s exposure, aperture (f stop) 7.46 field of view 190 mm, 1 × 1 binning, unfiltered emission. X-ray images were obtained with 10 s exposure, aperture (f stop) 2.8, field of view 190 mm, and 2 × 2 binning.

### Histology and Immunofluorescence

Skin tissue was fixed in 10 % neutral buffered formalin, paraffin embedded and sectioned (5 μm). Hematoxylin and eosin stained sections were imaged using a Nikon Eclipse Ti microscope, Plan Fluor ELWD 20X DIC L objective, DS-Fi2-U3 camera, and NIS elements software. Immunofluorescence antigen retrieval was performed in citrate buffer pH 6, boiling for 2 min and allowing to cool to room temperature. The sections were blocked with 2 % bovine serum albumin in PBS for 30 min at room temperature. Polyclonal rabbit anti-TEM8 (ab19387) and polyclonal goat anti-CMG2 (ab101711) were diluted 1:500 and incubated on the tissue overnight at 4°C. The sections were washed with PBS and incubated with anti-rabbit CF488 (Sigma) and anti-goat CF555 (Sigma) secondary antibodies for 30 min at room temperature. The sections were then washed with PBS and stained with DAPI. Immunofluorescence images were taken using an Olympus FV1200 confocal microscope, UPLSAPO20X objective, photomultiplier tube detector, and FV10 software.

### Colocalization Analysis

Quantification of fluorescence colocalization by Pearson's correlation coefficient (36) and intensity correlation quotient (37) was determined using ImageJ software (38, 39). Immunofluorescence images from 6 randomly selected sites within an SCC tumor were used for analysis. For each of the immunofluorescence images, a region containing no tissue was used to perform a background subtraction prior to analysis.

### Statistical Analysis

All statistical analysis was performed using GraphPad Prism software. Integrated density of *in vitro* internalization was analyzed using two tailed unpaired *T*-test with Welch's correction. Mean relative fluorescence intensity between the tumor and major organs within the UV^+^ group (*n* = 5) was analyzed using an ordinary One-Way ANOVA with Tukey's multiple comparisons test. Mean fluorescence intensity of the major organs between the UV^+^ (*n* = 5) and UV^−^ (*n* = 6) groups was analyzed using an ordinary Two-Way ANOVA with Sidak's multiple comparisons test.

## Results

### Fluorescently Labeled PA Maintains Cell Binding and Internalization *in vitro*

**Figure 1:**
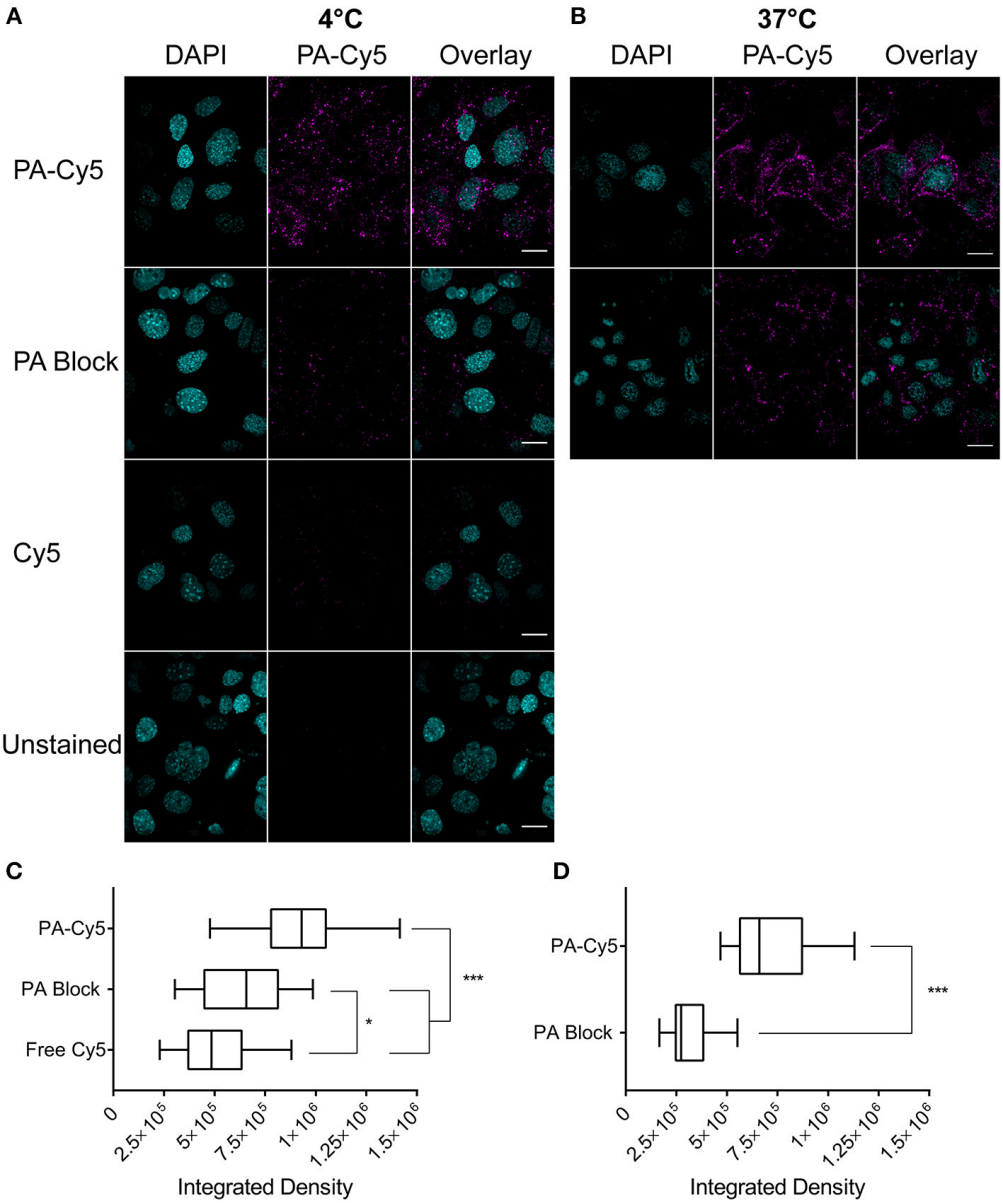
Fluorescently labeled PA maintains cell binding and internalization *in vitro*. Cells were plated on glass cover slips coated with poly-L lysine at 10^5^ cells/coverslip 18 h before staining. Cells were incubated with PA at 4°C to saturate the available receptor sites prior to internalization **(A)** or at 37°C for continuous cell uptake **(B)**. Blocking experiments (PA Block) were performed by incubating the cells with unlabeled PA on ice prior to the addition of PA-Cy5. Cells were washed with PBS before fixation in 4 % paraformaldehyde. Analysis of integrated density of Cy5 fluorescence per cell, visualized with box-and-whisker plots, identified PA-Cy5 internalization into SCC cells was significantly reduced by blocking the available receptor sites with unlabeled PA at 4°C **(C)** and 37°C **(D)**. Cyan, DAPI nuclear stain; magenta Cy5. Scale bar represents 20 μm. Statistical analysis performed using two tailed unpaired *T*-test with Welch's correction (^*^p = 0.0495, ^***^p < 0.0001).

We first confirmed that the amine bioconjuagted PA maintained binding and translocation into SCC cells. PA-Cy5 was incubated with cells at 4°C to saturate the available receptors and minimize cellular metabolism and receptor internalization (Figure 1A). Unbound PA-Cy5 was removed and the cells were incubated at 37°C to restore cell metabolism. Abundant fluorescence uptake was observed in the cells, with characteristic focal points of internalization. To confirm that the internalization of PA-Cy5 was a receptor mediated process, a blocking experiment was performed where the cells were pre-treated with unlabeled PA. Blocking the available receptors prior to incubation with PA-Cy5 resulted in a significant reduction of fluorescence (*p* < 0.0001, two tailed unpaired *T*-test), determined by analyzing the integrated density per cell (Figure 1C). The minimal fluorescence uptake observed in the 4°C PA block experiment was likely due to displacement of the unlabeled PA during PA-Cy5 incubation or natural oligomerization of PA/PA-Cy5 molecules. Continuous uptake of PA-Cy5 at 37°C resulted in greater distribution throughout the cells and more defined cell boundaries (Figure 1B). Again, blocking the available receptors prior to incubation with PA-Cy5 reduced the observable fluorescence resulting in a significant reduction (*p* < 0.0001, two tailed unpaired *T*-test) in integrated density per cell (Figure 1D). A control experiment was performed by incubating the cells with free Cy5 dye to determine non-specific binding or internalization due to the dye molecule, the low fluorescence uptake observed was likely due to pinocytosis.

### UV Irradiated Skin of the HPV38E6E7-FVB Model Shows Characteristics of SCC Development

**Figure 2:**
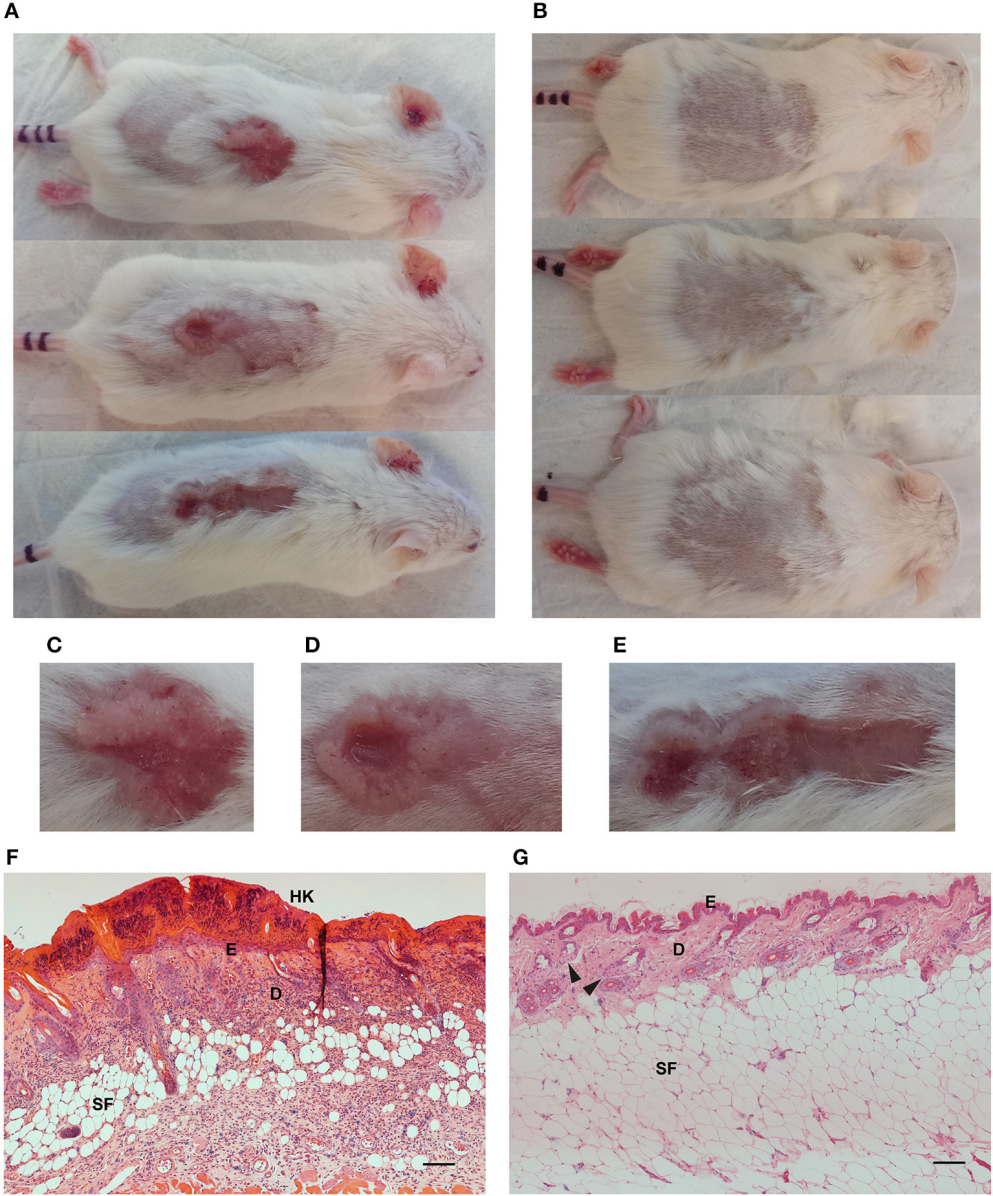
Images and skin histology of the HPV38E6E7-FVB mouse model demonstrating tumor development. Representative images of UV^+^ mice bearing tumors **(A)** and UV^−^ control mice **(B)**. Images were taken immediately prior to optical imaging. Tumors demonstrate characteristics associated with cutaneous SCC including open sores, red scaly patches, wart-like growth, and elevated growth with a central depression **(C–E)**. Hematoxylin and eosin **(H,E)** stained sections from a UV^+^ mouse SCC tumor **(F)** and dorsal skin from a UV^−^ control mouse **(G)**. HK, Hyperkeratosis; E, Epidermis; D, Dermis; SF, Subcutaneous fat; Black arrows highlight sebaceous glands. Scale bar represents 100 μm.

HPV38E6E7 transgenic animals exposed to UV irradiation for ~20 weeks developed skin lesions (Figure 2A) compared to healthy controls (Figure 2B). UV exposed animal's demonstrated red scaly patches, wart-like growths, open sores, and raised growths with a central depression characteristic of cutaneous SCC development (Figures 2C–E). Non-transgenic FVB mice do not develop skin lesions in response to response to repeated UV irradiation over the same time period (data not shown). Hematoxylin and eosin staining of formalin-fixed paraffin-embedded UV^+^ (Figure 2F) and UV^−^ (Figure 2G) skin samples showed significant differences associated with the development of SCC. UV^+^ tumor skin showed hyperkeratosis and parakeratosis characterized by the thickening of the stratum corneum, the outermost layer of the epidermis, and retention of nuclei in the stratum corneum, respectively. Epidermal hyperplasia and atypical crowding of keratinocytes was visible in the UV^+^ skin as well as budding of the basal layer of the epidermis into the dermis. Solar elastosis, increased vascularization, immune filtration, dense nucleation of the dermis, and invasion into the subcutaneous fat was also present in the UV^+^ skin.

### PA Is Highly Specific to Cutaneous SCC *in vivo*

**Figure 3:**
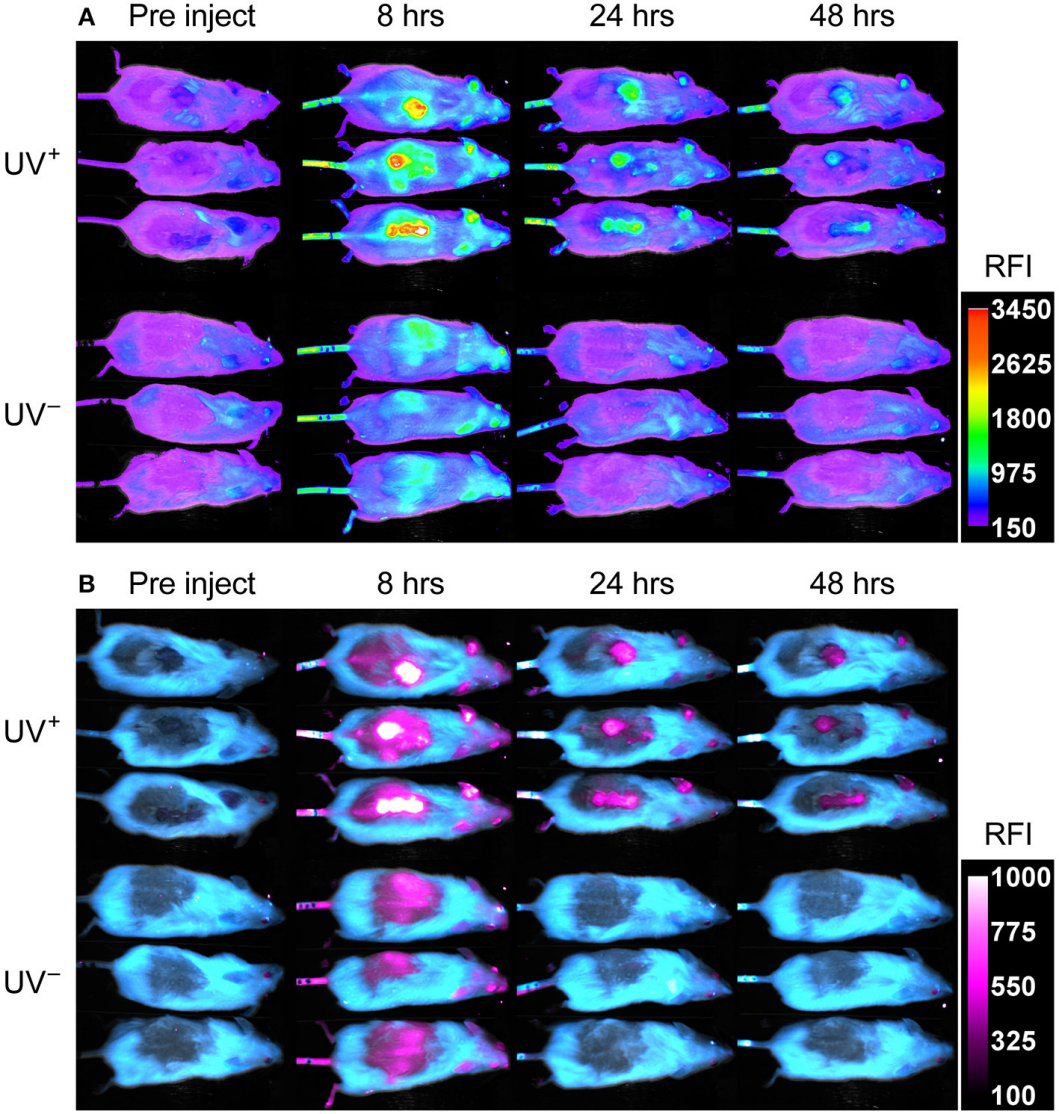
PA-Cy5 is highly specific to cutaneous SCC *in vivo*. Two groups of HPV38E6E7-FVB mice were imaged prior to injection, 8, 24, and 48 h post injection of 75 μg PA-Cy5 via tail vein. The UV^+^ group has developed squamous cell carcinomas on their back. The UV^−^ group is of a similar age to the UV^+^ group but have not been exposed to UV irradiation. Fluorescent *in vivo* optical images of PA-Cy5 using 630 nm excitation/700 nm emission **(A)**. Localization of PA-Cy5 to the SCC tumors is observed reaching peak relative fluorescence intensity (RFI) ~8 h post injection. Exclusive detection of tumors can be observed by 24 h post injection, where control animals reach background levels of fluorescence. Spectrally unmixed *in vivo* optical images **(B)**. A series of images were taken at each time point using multiple excitation wavelengths, measuring the emission at 700 nm. Optical spectra were modeled for fur auto-fluorescence and PA-Cy5 fluorescence using Bruker Multispectral software. These models were applied to the images, isolating the signal from the fur (cyan) and PA-Cy5 (magenta), demonstrating the tumor specific localization of PA-Cy5. Fluorescence arising from PA-Cy5 is undetectable in the skin 24 h post injection, but remains localized to the tumor 48 h post injection. The calibration bars represent relative fluorescence intensity (RFI). Fluorescence data is overlaid on reflectance (white light) images.

UV^+^ tumor bearing mice and UV^−^ naïve controls, were administered 75 μg fluorescent PA-Cy5 via the tail vein and optically imaged at several time points over 48 h. Imaging was performed 3 weeks after the final UV treatment to minimize any effects of UV induced inflammation. Fluorescence images were obtained using an excitation wavelength of 630 nm and emission wavelength of 700 nm as these were the optimal settings for visualization of Cy5 (Figure 3A). Fluorescence was localized to the regions which displayed SCC characteristics on the dorsal region and ears of UV^+^ mice, while no specific localization was visible in the UV^−^ mice.

To better visualize PA-Cy5 fluorescence, spectral unmixing was performed using Bruker Multispectral software. Images were obtained at every time point using seven excitation wavelengths (550, 570, 590, 600, 610, 620, and 630 nm) measuring the emission of each excitation at 700 nm. Spectral profiles were modeled for fur and PA-Cy5 fluorescence. The models were applied to all time points, separating the raw fluorescence signal into either fur or PA-Cy5 channels (Figure 3B). PA-Cy5 fluorescence correlates to the region of peak intensity observable in the initial fluorescence images. Unmixed images confirmed PA-Cy5 fluorescence localized to the tumor regions at 8 h post injection with lower fluorescence observable in the skin surrounding the tumor, consistent with the distribution of fluorescence seen in the 630 nm optical image. At 24 and 48 h post injection the PA-Cy5 fluorescence is highly specific to the tumor regions with no detectable signal in the tumor adjacent skin or control animals.

### PA-Cy5 Tumor Uptake Is Significantly Higher Than Healthy Tissues

**Figure 4:**
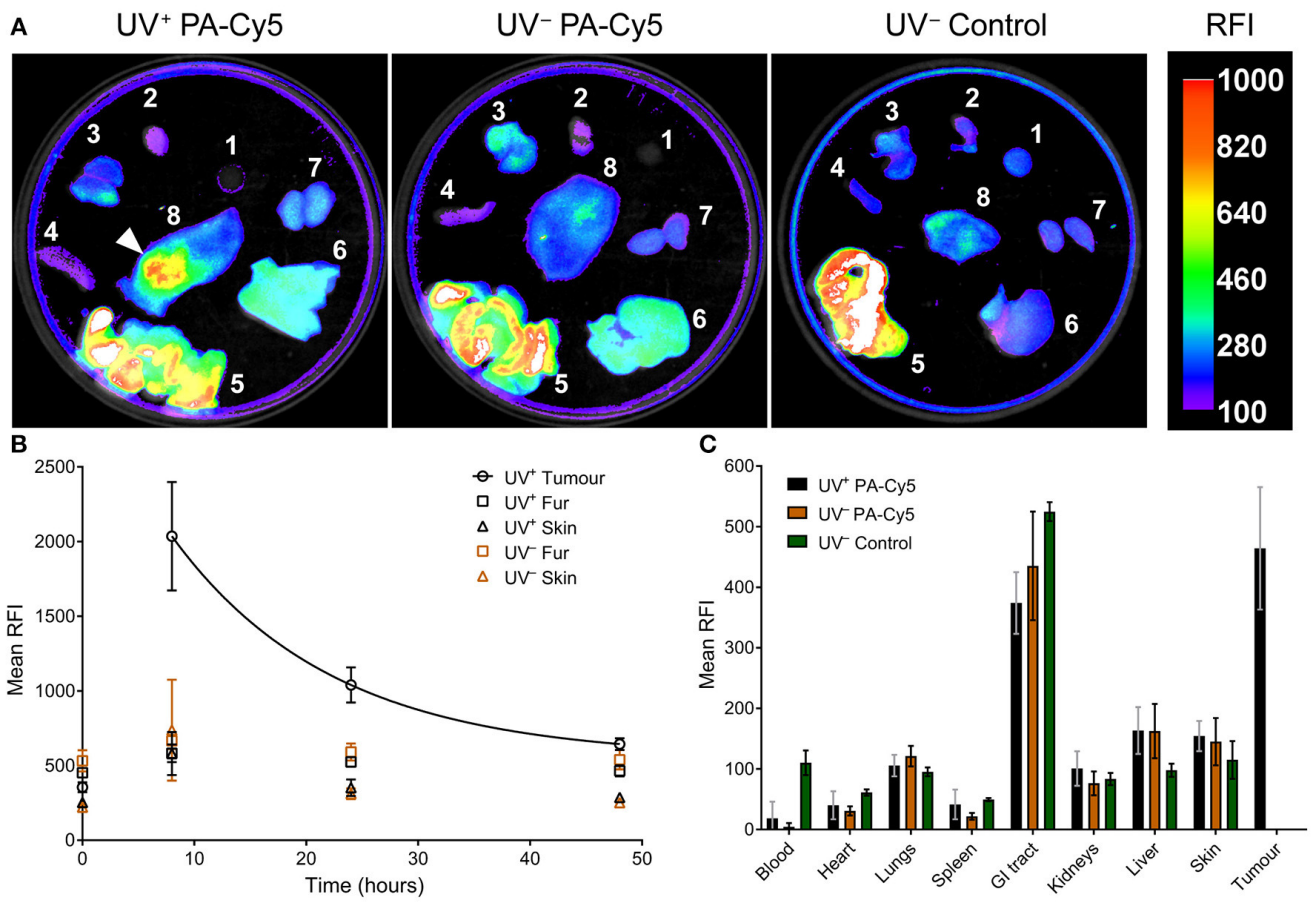
PA-Cy5 tumor uptake is significantly higher than healthy tissues. UV^+^ tumor bearing and UV^−^ control mice were euthanized 48 h post injection of PA-Cy5. The major organs from each animal were optically imaged using 630 nm excitation and 700 nm emission **(A)**. Organs were also imaged from UV^−^ mice that were not administered PA-Cy5 (UV^−^ Control) for autofluorescence comparison. 1. Blood, 2. Heart, 3. Lungs, 4. Spleen, 5. GI tract, 6. Liver, 7. Kidneys, and 8. Dorsal skin. A white arrow indicates the tumor region. Calibration bar represents relative fluorescence intensity (RFI). Fluorescence data is overlaid on X-ray images. Tumor, fur, and skin regions of interest were identified to determine mean RFI for UV^+^ HPV38E6E7-FVB tumor bearing mice (*n* = 5) and UV^−^ HPV38E6E7-FVB control mice (*n* = 6) intravenously injected with PA-Cy5 **(B)**. The mean RFI is plotted against time, with an exponential decay curve fit to the tumor data (solid line). Regions of interest were identified using reflectance images (white light) to prevent fluorescence bias. Error bars represent the standard deviations of the mean. 48 h after the injection of PA-Cy5 the animals were sacrificed, tumors and organs were removed and optically imaged **(C)**. Columns represent the mean RFI and the error bars represent standard deviations. One-Way ANOVA analysis resulted in significant differences (*P* < 0.0001) between the tumor and blood, heart, lungs, spleen, kidneys, liver, and skin within the UV^+^ PA-Cy5 group. Two-way ANOVA analysis resulted in no statistically significant differences between the UV^+^ PA-Cy5 and UV^−^ PA-Cy5 blood, heart, lungs, spleen, GI tract, kidneys, liver, or skin fluorescence.

Optical imaging was performed on the dissected mouse organs 48 h post injection of PA-Cy5 to identify fluorescence localization to the tumor and organs (Figure 4A). The highest relative fluorescence intensity (RFI) was observable at the tumor and in the gastrointestinal (GI) tract. The majority of fluorescence observed in the GI tract is a result of the animal feed, images acquired from organs of UV^−^ mice that did not receive PA-Cy5 resulted in similar mean RFI of the GI tract as in the PA-Cy5 administered mice. The fluorescence of the tumor corresponds to the skin abnormalities visible in the reflectance (white light) image and increased tissue density in the X-ray image. Fluorescence was also present in the liver, kidneys, and lungs at lower intensities.

Peak RFI was observed 8 h post injection, where mean RFI of the tumors was approximately three times higher than adjacent skin and control animals. Rapid clearance of fluorescence from non-tumor regions was observed. Twenty four hours post injection the control animals returned to pre-injection baseline, while the tumor bearing animals retained fluorescence exclusively at the tumor regions. The tumor localized fluorescence decreased over the imaging experiment, decaying exponentially at a rate of ~7 % mean RFI per hour (Figure 4B), and remained detectable 48 h post injection. The mean RFI of the tumor region was significantly different compared to the blood, heart, spleen, lungs, kidneys, liver, and skin (*p* < 0.0001, One-Way ANOVA) of the UV^+^ group, but not significantly different compared to the GI tract (*p* 0.064, One-Way ANOVA). The mean RFI of tumors was approximately three times higher than the liver and four times higher than the kidneys (Figure 4C). Organ fluorescence was comparable between the UV^+^ and UV^−^ groups administered PA-Cy5 with no statistically significant variance between the mean intensity of the blood, heart, spleen, lungs, kidneys, liver, or skin (*p* > 0.915, Two-Way ANOVA).

### PA-Cy5 Colocalizes With the Overexpression of TEM8 in SCC

**Figure 5:**
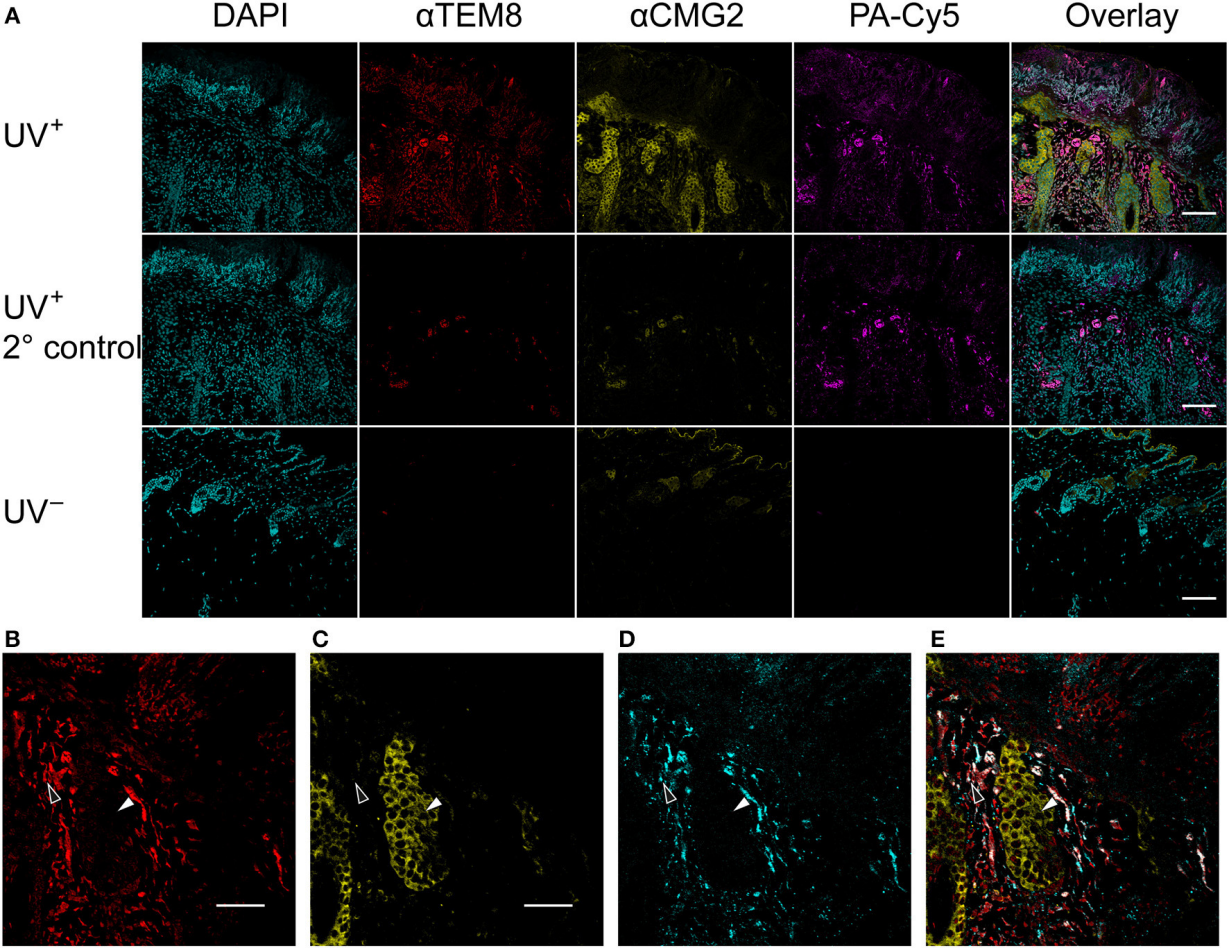
PA-Cy5 colocalizes with the overexpression of TEM8 in SCC. Skin samples were taken from tumor bearing mice (UV^+^) and control mice (UV^−^) 48 h after PA-Cy5 injection. Formalin-fixed paraffin-embedded skin samples were stained for the presence of the anthrax toxin receptors TEM8 and CMG2 using antibodies **(A)**. TEM8 was significantly overexpressed in tumor tissue compared to UV^−^ control, colocalized with the *in vivo* administered PA-Cy5. CMG2 was also significantly overexpressed in tumor tissue, with detectable expression in the UV^−^ control skin epidermis and sebaceous glands. Secondary antibody controls were performed using UV^+^ skin tissue. Representative single channel images of TEM8 **(B)**, CMG2 **(C)**, PA-Cy5 **(D)**, and three channel overlay **(E)** demonstrating differences in fluorescence localization within the skin tissue. Black arrows highlight an epidermal budding region with low TEM8 and PA-Cy5 fluorescence, high CMG2 fluorescence. White arrows highlight a dermal region with high TEM8 and PA-Cy5 fluorescence, low CMG2 fluorescence. DAPI, 405 nm; TEM8, 488 nm; CMG2, 555 nm; PA-Cy5, 630 nm. Primary antibodies were incubated on the tissue for 1 h at room temperature; secondary antibodies for 30 min at room temperature. Scale bar represents 100 μm.

Anti-TEM8 and anti-CMG2 antibodies were used to visualize the expression of the anthrax toxin receptors in formalin-fixed paraffin-embedded mouse skin tissue (Figure 5A). TEM8 expression was significantly upregulated throughout the UV^+^ skin, localized to the nucleated stratum corneum and dermal regions. No detectable expression of TEM8 was visible in the UV^−^ control skin. CMG2 was also upregulated in the UV^+^ skin compared to UV^−^ skin, with the highest expression localized to the thickened epidermis and basal budding structures. Lower expression of CMG2 was observed in the UV^−^ skin localized to the epidermis and sebaceous glands. Both TEM8 and CMG2 secondary antibody controls showed non-specific binding that appears localized to vessel structures. PA-Cy5, administered to the animals via tail vein 48 h prior to culling, was present in the UV^+^ skin but not the UV^−^ skin consistent with the optical imaging experiments. PA-Cy5 was highly colocalized with TEM8 expression in the dermal regions of the skin tissue but was not detectable in the regions with the highest CMG2 expression such as the epidermal budding structures (Figures 5B–E). Colocalization analysis was performed on 6 randomly selected regions of an SCC tumor (Table 1), quantified using Pearson's correlation coefficient (PCC) and intensity correlation quotient (ICQ). PCC is a measure of linear relationship between pixel signal co-occurrence and intensity resulting in a value from −1 (perfect negative correlation) to +1 (perfect positive correlation), with distributions that are uncorrelated resulting in values near zero (40). ICQ is a measure of correlation based on the ratio of pixel intensity synchronicity between two channels with respect to mean image intensities resulting in a value from−0.5 (perfect exclusive) to + 0.5 (perfect colocalization), where values near zero represent random staining (37). Across all samples tested, the correlation between TEM8 and PA-Cy5 is consistently higher than CMG2 and PA-Cy5 for both PCC and ICQ methods.

**Table 1:** Colocalization quantification of an intravenously injected PA-Cy5 with its receptors in an SCC tumor.

**Sample**	**Pearson's correlation coefficient**		**Intensity correlation quotient**	
	**TEM8 & PA-Cy5**	**CMG2 & PA-Cy5**	**TEM8 & PA-Cy5**	**CMG2 & PA-Cy5**
1	0.389	0.077	0.117	0.069
2	0.651	0.276	0.253	0.17
3	0.555	0.239	0.202	0.155
4	0.663	0.355	0.29	0.222
5	0.708	0.292	0.288	0.175
6	0.648	0.141	0.25	0.123

## Discussion

Optical imaging is a potential cost effective means of screening superficial tumors in preclinical models (41). Optical probes that target skin cancers could have significant benefit in skin cancer screening, improving tumor detection and reducing costs. We have identified that PA, a protein produced by *B. anthracis*, conjugated to a fluorescent dye is highly specific to HPV positive UV induced mouse SCC. Although there is overexpression of both anthrax toxin receptors, the administered PA-Cy5 is primarily colocalized with TEM8.

Amine bioconjugation is a common technique for labeling proteins of interest with imaging molecules. A limitation of non-specifically labeling PA with an amine reactive cyanine dye is that we were unable to spectrally quantify the degree of labeling. A known occurrence with cyanine dyes is their propensity to form aggregates when in close proximity, resulting in a homo-FRET self-quenching effect (42). By using the sulfonated analog of Cy5 we avoid the homo-fret self-quenching (42, 43), but due to a high degree of labeling there is a non-linear relationship between degree of labeling and spectral properties. For the purposes of assessing the localization of PA-Cy5 to SCC tumors the degree of labeling is inconsequential, provided that PA-Cy5 maintains its ability to bind and internalize into cells.

As there is potential to disrupt protein stability or binding affinity by non-specifically modifying amines, we first confirmed that the PA-Cy5 protein maintained cell binding and internalization *in vitro*. Receptor specific uptake of PA-Cy5 into SCC cells was observed, which was drastically reduced by pre-incubation with unlabeled PA, demonstrating that non-specific modification of PA amines does not prevent receptor binding or internalization. Receptor specific uptake of PA-Cy5 into SCC cells was observed, which was drastically reduced by pre-incubation with unlabeled PA, demonstrating that non-specific modification of PA amines does not prevent receptor binding or internalization. By reducing the temperature cellular metabolism and receptor turnover is reduced (44). Incubated the cells at 4°C with PA-Cy5 and removing unbound protein before increasing the temperature demonstrates that PA-Cy5 is associated with the cells. Subsequent internalization of PA-Cy5 was identified by focal points of fluorescence within the cell. As we do not see a clear delineation of the cell membrane it is likely that the PA-Cy5 association id due to receptor binding, and is not a result of non-specific cell membrane association. Pre-incubation with unlabeled PA significantly reduces the cellular uptake of fluorescence (*p* < 0.0001, two tailed unpaired *T*-test with Welch's correction), further demonstrating a receptor specific interaction between PA-Cy5 and the SCC cells. Continuous internalization of PA-Cy5 at 37°C demonstrated internalized fluorescence as well as defined cell surfaces, indicating cell surface associated PA-Cy5 at the time of fixation. Pre-incubation with unlabeled PA again significantly reduces the cellular uptake of fluorescence (*p* < 0.0001, two tailed unpaired *T*-test with Welch's correction).

The fluorescence observed in the PA blocking experiments may be due to several factors. Firstly, at 4°C the cells have reduced metabolism, but some degree of receptor recycling still occurs (44). This may be enhanced by PA activated receptor internalization (45) and an equilibrium between surfaced exposed receptors and intracellular receptors (46). Secondly, as PA is able to form complexes with itself (47) PA-Cy5 may associate with unlabeled PA bound to receptors. In order to form these complexes PA requires enzymatic cleavage (48), which would also occur at a reduced rate due to the low temperature, and therefore would limit the effect. It is important to note that it is unknown whether the amine modification has disrupted this aspect of PA. Thirdly, the binding of PA-Cy5 is occurring in a dynamic system. Although the available receptors may be initially occupied by unlabeled PA, in the presence of excess PA-Cy5 there may be some dissociation of unlabeled PA allowing for the association of PA-Cy5.

As the sulfonated analog of Cy5 used here is cell impermeable due to its anionic charge (49), the free Cy5 experiment demonstrates that there is negligible dye uptake by the SCC cells through non-specific internalization. This experiment further validates that the cellular uptake of fluorescence is a direct result of PA and its receptor specific internalization.

PA-Cy5 administered via tail vein resulted in rapid tumor uptake, reaching maximum tumor intensity 8 h post injection and tumor specific localization by 24 h. The time scale of PA-Cy5 tumor uptake and clearance observed here is significantly longer than the clearance reported in the literature for wild type PA (50). The increase in circulation time may be due to reduced proteolysis caused by steric hindrance of protease recognition sites, or changes in protein electrostatic and hydrophobic properties as a result of dye conjugation. The highly specific and prolonged retention of the intravenously administered PA-Cy5 to the SCC tumors, on both the dorsal skin and ears, allows for a practical imaging time window that may translate clinically. Light scattering, light attenuation, and autofluorescence are potential limitations of fluorescence optical imaging. This model of SCC is extremely well-suited to optical studies due to the shallow tumor depth, limiting any effect of light scattering and attenuation. Additionally, the similarity in tumor development between this model and human cutaneous SCC could improve clinical translation. While the spectral unmixing can be used to better visualize specific fluorophores from endogenous signals and autofluorescence, using near infra-red fluorophores that are excited by longer wavelengths would also improve visualization (51).

*Ex vivo* imaging was used to obtain semi-quantitative data on the biodistribution of fluorescence within the major mouse organs. Forty-eight hours post injection, relatively low fluorescence intensity was observed in the lungs, kidneys, liver, and healthy skin. Significantly higher fluorescence intensity was observed in the GI tract and tumor. Whilst the majority of fluorescence within the GI tract can be attributed to the mouse feed (52), the significant localization of fluorescence to the tumor suggests specific upregulation of the anthrax toxin receptors. Although TEM8 has been suggested to be highly expressed in the epithelial cells of the skin, lungs, and intestine (53), our results demonstrate the natural expression of the anthrax toxin receptors in healthy tissues results in significantly lower uptake of PA-Cy5 compared to tumor regions.

We identified that both anthrax toxin receptors are overexpressed in the UV^+^ skin associated with SCC relative to the UV^−^ naïve control. Interestingly, TEM8 and CMG2 are expressed in different cellular structures within the skin. TEM8 expression was localized to the nucleated stratum corneum and dermis, whereas CMG2 expression was primarily localized to the thickened epidermis including basal budding structures but also present at lower levels throughout the dermis. In the UV^−^ skin TEM8 was undetectable, whereas CMG2 expression was detectable selectively in the epidermis and sebaceous glands. To date there is limited information about the tissue distribution of TEM8 and CMG2. Published immunofluorescence of CMG2 expression in healthy mouse skin resulted in highly similar distribution (12), confirming our results. The TEM8 expression we observed, however, does not support published results that describe localization to the keratinocytes (epidermis) and follicular cells of healthy mouse skin (53). Interestingly, the previously reported TEM8 expression patterns are highly similar to the CMG2 expression observed here. It is possible that the polyclonal antibody used for the previous immunohistochemistry study inadvertently cross reacted with CMG2. As we are the first to co-stain for both receptors we can be confident that the observed distribution presented here is attributed to the individual anthrax toxin receptors.

The expression pattern of the anthrax toxin receptors is consistent with the localization of their endogenous ligands. TEM8 was previously identified to interact with collagen I (15) and collagen α3 VI (16). Collagen I is the most abundant collagen subtype of the ECM and is the major component of the dermal interstitial matrix, collagen α3 VI is similarly located in the interstitial matrix (54). CMG2 is known to bind laminin, collagen IV (23) and collagen VI (24). Laminin and collagen IV are ECM components primarily located in the basal lamina and basement membrane anchoring cells to connective tissue (54), such as the epidermis to the dermis. Although the function of the anthrax toxin receptors during tumor development is still unknown, ECM interactions are important in many cellular functions that drive tumor development, invasion, and metastatic progression (55). The anthrax toxin receptors may inform on tumor specific ECM changes providing valuable information on disease progression and metastatic potential.

The intravenously injected PA-Cy5 was highly colocalized with TEM8 but not present in the regions of the highest CMG2 expression. The reason for selectivity to TEM8 in this case is unknown. CMG2 has been described as the primary receptor for the anthrax toxins due to higher PA affinity (56). *In vivo* it is possible that TEM8 may be more accessible than CMG2 from an intravenous injection. Immunofluorescence demonstrates that TEM8 is generally expressed within the dermis, while CMG2 is present in both dermal and epidermal regions. As the majority of skin microvasculature is located within the dermis (57), receptors expressed in this region are more accessible to an intravenously administered agent. The thickened epidermis likely suffers from low or no blood flow, as a result the administered PA-Cy5 is unable to reach the epidermal regions despite significant expression of CMG2. In addition the available CMG2 receptors in the epidermal regions may also be occupied by endogenous ligands preventing PA interaction.

Quantification of colocalization consistently demonstrated greater colocalization of the intravenously administered PA-Cy5 with TEM8 expression compared to CMG2 expression. It is important to note that due to the dependence on a linear relationship the Pearson's correlation coefficient is affected by the heterogeneity of receptor expression and PA-Cy5 uptake within the tissue, as a result the values determined are under-representative of the degree of correlation (40). Similarly ICQ is also under-represented in this data. As ICQ is a measure of the ratio of pixel intensity synchronicity between the receptor and PA-Cy5, there will always be asynchronicity as a result of PA-Cy5 binding to two receptors. Despite these limitations, the PCC and ICQ values can still be directly compared within each sample and consistently demonstrates that there is a higher degree of correlation between TEM8 and PA-Cy5 relative to CMG2 and PA-Cy5.

The specific localization and prolonged retention of PA-Cy5 to tumors is likely due to the mechanism of receptor internalization. TEM8 is regulated by an endocytic transport mechanism, where there is an equilibrium between surface and cytosolic TEM8 (46). This pool of available unoccupied receptors residing in the endosomal recycling pathway allows rapid PA uptake by continually replacing surface receptors (46). PA is also able to activate receptor clustering (58) and endocytosis (45) similar to endogenous ligands, in addition to forming heptameric or octameric complexes (59), further enhancing cellular uptake. Healthy cells that have relatively low or undetectable levels of TEM8 on their surface will be unable to internalize PA at the same rate, providing the necessary contrast for tumor detection. The cell surface expression, recycling mechanism, as well as the potential role in ECM remodeling and invasion, highlight TEM8 as a novel biomarker of cutaneous SCC for targeted imaging. While the oligomerization and active internalization of PA is a unique advantage over antibody or peptide based targeted agents for rapid and selective tumor uptake.

PA labeled with a fluorescent imaging agent may provide rapid visual identification of lesions in a clinical setting, particularly with patients who have a personal history or are at high risk of developing SCC, reducing time and costs associated with patient screening. As surgical resection is the most common treatment for cutaneous SCC and its precursor lesions, a PA based imaging agent may also aid tumor removal by informing on lesion boundaries as defined by the overexpression of the anthrax toxin receptors. In addition to diagnosis, PA could be further developed as a drug conjugate for targeted systemic treatment of SCC. PA as a targeting agent may be particularly useful for patients who require treatment of many tumors or as an alternative treatment option for patients who are poor candidates for surgical resection.

Overall we show that intravenously injected recombinant *B. anthracis* PA labeled with a near infrared fluorophore is highly specific to a physiologically relevant mouse model of cutaneous SCC. Whilst there is overexpression of both anthrax toxin receptors, TEM8 appears to have a more significant role in the uptake of the intravenously administered PA-Cy5. As TEM8 is extremely conserved between humans and mice, there is potential for direct translation. Based on these results, PA is a highly specific agent for targeting tumors overexpressing the anthrax toxin receptors, such as cutaneous SCC, and may have future diagnostic or therapeutic applications as a systemically administered targeting agent.

## Data Availability

The raw data supporting the conclusions of this manuscript will be made available by the authors, without undue reservation, to any qualified researcher.

## Author Contributions

TC, YT, MM, and IB: conception and design; TC, NF, JW, MV, JG, and NP: development of methodology; TC, NF, MV, JG, and NP: acquisition of data; TC: analysis and interpretation of data; TC, YT, MM, IB, JW, and NF: writing, review and/or revision of the manuscript; YT, MM, IB, JW, and NF: study supervision.

### Conflict of Interest Statement

The authors declare that the research was conducted in the absence of any commercial or financial relationships that could be construed as a potential conflict of interest.
